# Digital Simulation–Based Ultrasound Training for Physiotherapy Students: Prospective Randomized Controlled Trial

**DOI:** 10.2196/87897

**Published:** 2026-07-02

**Authors:** Samuel Fernández-Carnero, Belen Diaz-Pulido, Jorge Méndez-Rodriguez, Daniel Pecos-Martin, Santiago Garcia-Miguel, Alexander Achalandabaso-Ochoa, Nicolas Cuenca-Zaldívar, Fermín Naranjo-Cinto Sr

**Affiliations:** 1 Universidad de Alcalá, Facultad de Medicina y Ciencias de la Salud,, Departamento de Enfermería y Fisioterapia, Grupo de Investigación Fisioterapia y Dolor, 28801 Alcalá de Henares, Spain; 2 Development and Programming Department, Amazonic Global, s.l, Madrid, Madrid, Spain; 3 Department of Health Sciences, Unversidad de Jaen, Jaen, Spain; 4 Research Group in Nursing and Health Care, Puerta de Hierro Health Research Institute—Segovia de Arana (IDIPHISA), C. Joaquín Rodrigo, 1, Majadahonda, Madrid, 28222, Spain, 34 639962935; 5 Facultad de Medicina, Benemerita Universidad Autonoma de Puebla, Puebla, Mexico

**Keywords:** ultrasound education, simulation-based training, physiotherapy education, digital health education, randomized controlled trial, item response theory

## Abstract

**Background:**

Ultrasound education has traditionally relied on on-site training, but scalable digital solutions are increasingly needed.

**Objective:**

This study aimed to evaluate the effectiveness of a simulation-based online ultrasound platform compared with traditional on-site training.

**Methods:**

A prospective randomized blinded study was conducted with 68 physiotherapy students (n=34 per group). Participants were assigned to a simulation-based online training platform (WAZO) or traditional on-site instruction. Both groups completed identical theoretical and practical assessments. Item response theory using a Rasch model was applied to evaluate item difficulty and student ability.

**Results:**

No significant differences were found between the online and on-site groups in theoretical scores (mean 4.94, SD 1.47 vs mean 5.08, SD 1.14; *P*=.65) or practical performance variables, including probe handling (26/34, 76.5% vs 28/34, 82.4%; *P*=.37) and structure identification (24/34, 70.6% vs 22/34, 64.7%; *P*=.19). Measurement outcomes also showed no significant differences, including structure diameter (mean 3.78, SD 0.79 mm vs mean 3.98, SD 1.27 mm; *P*=.46) and structure surface distance (mean 3.94, SD 1.97 mm vs mean 3.24, SD 0.64 mm; *P*=.06). Item response theory analysis identified image optimization, structure diameter, and structure surface distance as the most difficult items, while probe handling and structure identification were the most informative. The model demonstrated high discriminative performance (area under the curve=0.93), with sensitivity of 87% and specificity of 80%.

**Conclusions:**

Simulation-based online ultrasound training provides comparable theoretical and practical outcomes to traditional on-site instruction, supporting its use as a scalable and accessible educational alternative.

## Introduction

Training in ultrasound for health science professionals for clinical use is based on the traditional classroom model [1] due to the empirical need to learn this tool, which has demonstrated its usefulness and suitability. Innovation in higher education is an opportunity to provide better educational experiences that allow health care professionals to provide a greater benefit to society [2]. Point-of-care ultrasound has demonstrated high reliability in soft-tissue scanning, being completely harmless, low cost, and high value for bedside use [3,4]. These conditions mean that ultrasound enjoys greater consideration than other imaging tests because the clinician can extract relevant information that can be directly correlated with clinical history, making diagnosis more powerful, because imaging tests are not always indicative of dysfunction or pain [5]. The training pathway in some health sciences disciplines includes ultrasound competencies from the second year of training [6], an approach that has also been adopted by other disciplines to better prepare professionals for clinical practice.

The use of simulators in certain disciplines such as aeronautics [7], automotive engineering, or robotic surgeries is widely accepted and proven to be a fundamental basis for a lower carbon footprint. Online training systems in the health sciences have been shown to enable professionals to achieve higher success rates, lower costs, and greater efficiency and to work more quickly [8].

Online and digital materials have been previously used to explore the possibility of ultrasound education in different specialties, such as obstetrics [9], anesthesia residents for lung assessment [10], physiotherapy [11,12], postdegree physician echocardiography [13], and other allied health sciences, demonstrating the potential of these resources and the need to improve this line of education to achieve the necessary skills and knowledge for clinical settings.

To the best of our knowledge, the current models of training in ultrasound are based on videos of varying complexity, leaving aside simulation and training in aspects of image optimization or the use of tools such as gain, dual image, or calipers.

Item response theory (IRT) was used to evaluate the psychometric properties of the assessment. Detailed methodological assumptions and model specifications are provided in Multimedia Appendix 1 [14,15].

One of the main arguments in favor of IRT methods is that they do not use, as in classical test theory, the standard error of measurement (SEM) of the tested population to estimate the parameters, so the calculated parameters are not influenced by the characteristics of the population. This allows the creation of questionnaires whose properties are invariant regardless of the population to which they are applied [16].

The use of the Rasch analysis requires the fulfillment of several assumptions: unidimensionality, that is, that all the items measure the same construct; local independence, that is, the items are not related to each other; and invariance of the parameters, that is, the items present the same item characteristic curves across subsamples of the participants. Finally, the existence of differential item functioning must be verified, that is, whether students with the same level of θ belonging to different subgroups of the sample have the same probability of responding correctly to the items [17].

The purpose of this study was to evaluate whether online training with a simulator platform called WAZO (WAZO Technologies Lab, sl) [18] (“the platform” hereafter) could be a viable alternative to traditional training, given that point-of-care ultrasound is becoming essential, and health care professionals need the knowledge necessary to provide better health care.

## Methods

### Overview

A prospective longitudinal experimental study was designed in which all second-year students enrolled in the Assessment in Physiotherapy course of the e Degree in Physiotherapy program at the University of Alcalá (Spain) were randomized in 2 groups: online (experimental) and on-site (control).

The study design followed the CONSORT (Consolidated Standards of Reporting Trials) standard [19]. This study was not prospectively registered in a clinical trials registry, as it was conducted as an educational intervention within an academic setting.

The inclusion criterion was successful completion of previous subjects related to this course (such as anatomy), and the exclusion criterion was the presence of vision and/or mobility difficulties in the upper limbs.

The on-site group received the basic musculoskeletal ultrasound training provided each year as part of the General Procedures II and Assessment in Physiotherapy courses through face-to-face lectures followed by practical sessions using ultrasound scanners at a ratio of 1 ultrasound scanner for every 2 students, with teacher support provided in small groups of 12 students. The online group was given access to the online ultrasound training platform without the opportunity to ask questions to instructors or handle an ultrasound scanner until 2 hours before the examination, when they were allowed contact with the device.

For the evaluation of the competencies acquired, all students took the same tests. The study variables were extracted from these tests, consisting of two parts: (1) theoretical (10 multiple-choice questions with 4 answers, only 1 of which was valid), and (2) practical examination to identify an anatomical structure (selected at random) using ultrasound in a partner (selected at random) where the examiner collected the following items on a rubric (success or failure, time to recognize it, position of the assessor, position of the patient, skill in handling the probe, and image optimization). Measurements in a training phantom (Venipuncture IV Injection Training Pad Model, VTurboWay, Ltd) of the intragel structures (structure diameter and structure surface distance) were taken with the ultrasound calipers.

The examiners who assessed the students were different from the investigator who performed the first randomization for online and on-site assignment to avoid bias at this point and differed from the instructor who taught the face-to-face classes. The phantom measurements were taken by an ultrasound expert on the same day prior to the student assessments to provide a reference standard for subsequent comparative analysis.

Statistical analysis was performed with R software (version 4.1.3: R Foundation for Statistical Computing). The significance level was set at *P*<.05. The distribution of the quantitative variables was tested with the Shapiro-Wilk test. Quantitative variables are expressed as mean (SD) and qualitative variables as absolute and relative values (%).

The presence of significant differences in the responses between the online and on-site groups was verified using Fisher exact test in the case of qualitative variables, and with the Mann-Whitney *U* test or 2-tailed Student *t* test for independent samples (after checking the assumption of homogeneity of variances with the Levene test) in the quantitative variables based on their distribution.

For structure diameter and structure surface distance measurements, the intraclass correlation coefficient (ICC [3,1]) was calculated between student measurements and the reference values using a 2-way mixed single-measure model (consistency and absolute agreement). Relative reliability was defined as poor (<0.5), moderate (0.5-0.75), good (0.75-0.9), or excellent (>0.9). As absolute reliability, the SEM was calculated. The minimum detectable change was also calculated.

IRT analysis using a Rasch model was conducted to estimate item difficulty and student ability. Detailed assumptions, model fit procedures, and statistical tests are provided in Multimedia Appendix 1.

To minimize potential bias related to conflicts of interest, several safeguards were implemented. Group allocation was performed by an independent researcher not involved in teaching or assessment, ensuring allocation concealment. Examiners responsible for evaluating both theoretical and practical performance were independent of the intervention and blinded to group assignment. These examiners were not involved in the development of the platform and had no access to allocation data.

All assessments were conducted under standardized conditions using identical evaluation criteria for both groups. Although participants could not be blinded due to the nature of the intervention, no explicit indicators of group allocation were provided to examiners during the evaluation process.

### Ethical Considerations

The study was approved by the Vicerrectorado de Innovación Docente from the Universidad de Alcalá (UAHEV/1434). An informed consent form was signed by all participants. In addition, data were analyzed after anonymization to further reduce the risk of bias.

## Results

### Overview

The sample consisted of 68 students, mostly women (n=42, 61.8%). No significant differences were observed in the demographic characteristics and examination outcomes between the groups (Table 1).

In the analysis of the structure diameter and structure surface distance vs reference measurements, the ICC (3,1) was not significant with poor values and high and precise SEMs (Table 2).

**Table 1.** Demographic characteristics and examination outcomes of the participants.

Variables	Overall (n=68)	Online (n=34)	On-site (n=34)	*P* value
Sex, n (%)				>.99
Female	42 (61.8)	21 (61.8)	21 (61.8)	
Male	26 (38.2)	13 (38.2)	13 (38.2)	
Patient position, n (%)				.37
Correct	56 (82.4)	27 (79.4)	29 (85.3)	
Incorrect	7 (10.3)	3 (8.8)	4 (11.8)	
Missing	5 (7.4)	4 (11.8)	1 (2.9)	
Sonographer position, n (%)				.24
Correct	62 (91.2)	30 (88.2)	32 (94.1)	
Incorrect	1 (1.5)	0 (0)	1 (2.9)	
Missing	5 (7.4)	4 (11.8)	1 (2.9)	
Probe handling, n (%)				.37
Correct	54 (79.4)	26 (76.5)	28 (82.4)	
Incorrect	9 (13.2)	4 (11.8)	5 (14.7)	
Missing	5 (7.4)	4 (11.8)	1 (2.9)	
Image optimization, n (%)				.31
Correct	17 (25)	7 (20.6)	10 (29.4)	
Incorrect	46 (67.6)	23 (67.6)	23 (67.6)	
Missing	5 (7.4)	4 (11.8)	1 (2.9)	
Structure identification, n (%)				.19
Correct	46 (67.6)	24 (70.6)	22 (64.7)	
Incorrect	17 (25)	6 (17.6)	11 (32.4)	
Missing	5 (7.4)	4 (11.8)	1 (2.9)	
Test duration (seconds), mean (SD)	101.44 (66.41)	102.52 (61.55)	100.46 (71.48)	.90
Structure diameter (mm), mean (SD)	3.88 (1.06)	3.78 (0.79)	3.98 (1.27)	.46
Structure surface distance (mm), mean (SD)	3.57 (1.47)	3.94 (1.97)	3.24 (0.64)	.06
Theoretical exam score, mean (SD)	5.01 (1.30)	4.94 (1.47)	5.08 (1.14)	.66

**Table 2.** Interobserver reliability between student and reference measurements.

Group and variable	ICC^a^ (95% CI)	Mean (SD; 95% CI)	SEM^b^ (95% CI)	MDC^c^
Online				
Structure diameter (mm)	0.253 (−0.052 to 0.514)	3.771 (0.606; 3.59-3.951)	0.537 (0.408-0.666)	1.488
Structure surface distance (mm)	0 (−0.301 to 0.301)	4.304 (1.453; 3.923-4.684)	1.457 (1.057-1.856)	4.038
On-site				
Structure diameter (mm)	0.009 (−0.278 to 0.295)	3.863 (0.923; 3.629-4.097)	0.923 (0.712-1.134)	2.558
Structure surface distance (mm)	0 (−0.287 to 0.287)	3.959 (0.921; 3.84-4.078)	0.67 (0.454-0.885)	1.857

a ICC: intraclass correlation coefficient.

b SEM: SE of measurement.

c MDC: minimal detectable change.

### IRT Analysis

#### Overview

**Figure 1.**
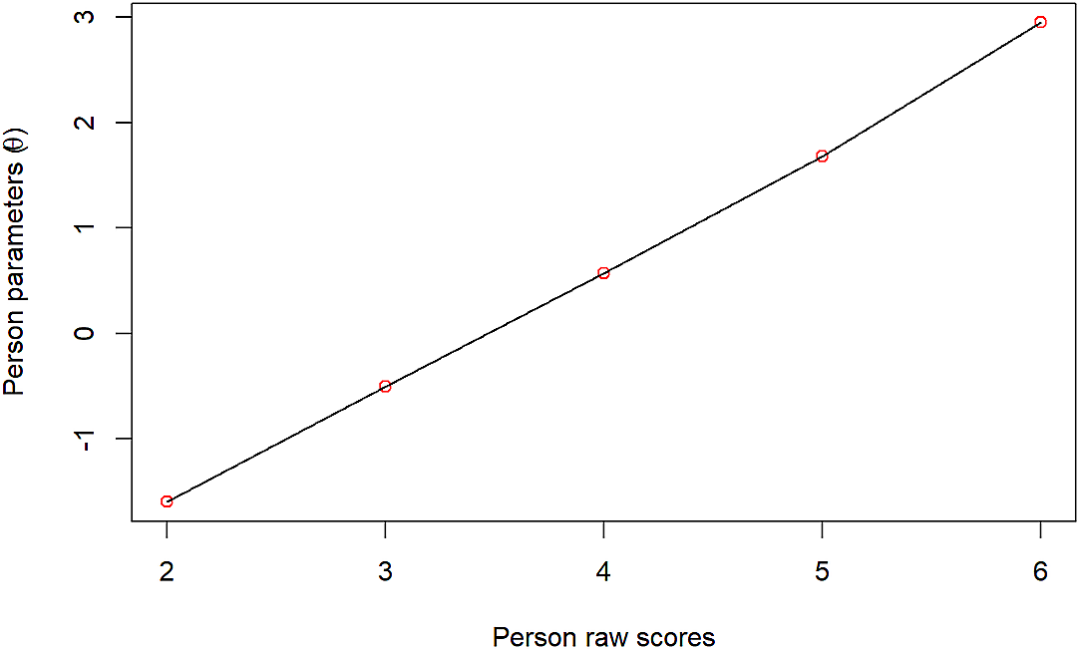
Plot of person parameters showing the monotonic mapping of total raw scores to the estimated latent ability parameters. Higher raw scores correspond to higher levels of latent competence, providing a linear-like transformation within the observed score range (2-6).

**Figure 2.**
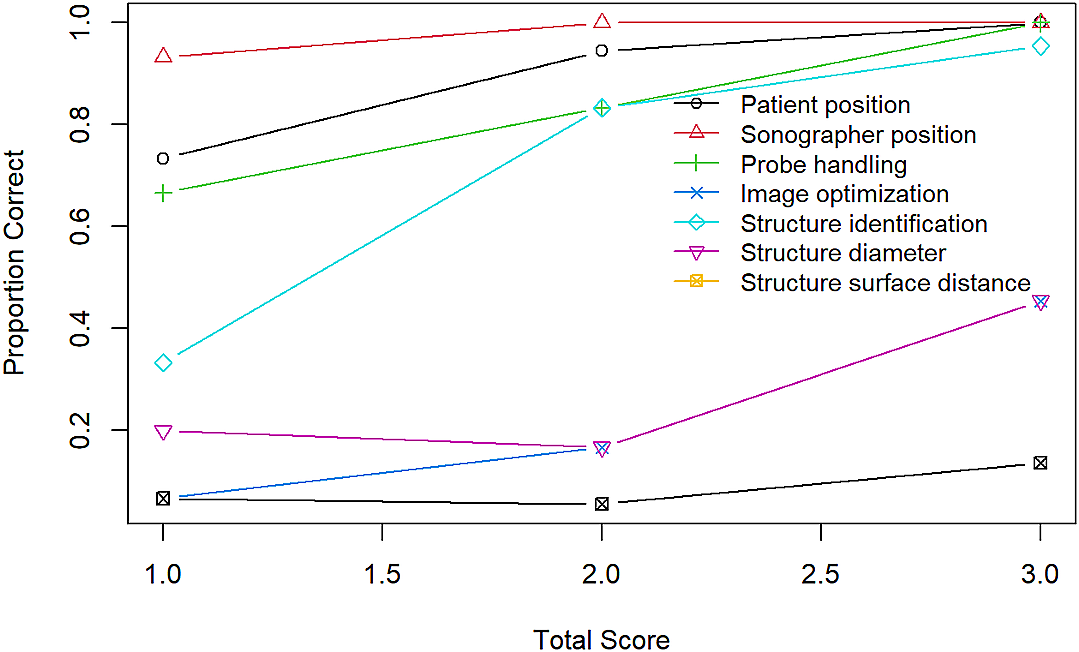
Test information plot showing the proportion of correct responses for each assessment criterion across overall score levels (1.0-3.0). Steeper lines (eg, structure identification) indicate criteria with high discriminative power. Lines near the top (eg, sonographer position) represent easier tasks, whereas lines near the bottom (eg, structure surface distance) represent highly difficult tasks across all proficiency levels.

The average level of success of the items was 59.637%, with the questions on image optimization, structure diameter, and structure surface distance having a hit rate of less than 50%. A total of 15 students answered 3 questions correctly, 18 answered 4 questions correctly, and 22 answered 5 questions correctly (Figure 1). Only 5 students answered 6 questions correctly and 3 students answered 2 questions correctly; there were no students who answered all the questions correctly or who failed all of them. It can be seen that in the questions on patient position, sonographer position, probe handling, image optimization, and structure identification, the proportion of correct answers increases as the final score obtained increased, while in the questions on structure diameter and structure surface distance, the proportion of hits decreased up to a score of 2 and then increased slightly up to the maximum score. The probability of correct responses increased consistently with total score for most items, supporting the expected hierarchical structure of the assessment (Figure 2).

#### Model Assumptions

**Figure 3.**
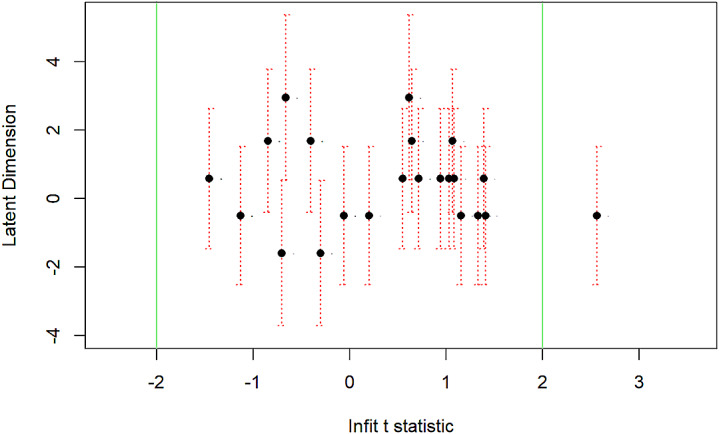
Person vs infit t-statistic plot displaying person ability parameters on the vertical axis and their respective infit t-statistics on the horizontal axis. The green vertical lines at −2 and 2 represent the thresholds for acceptable item fit.

**Figure 4.**
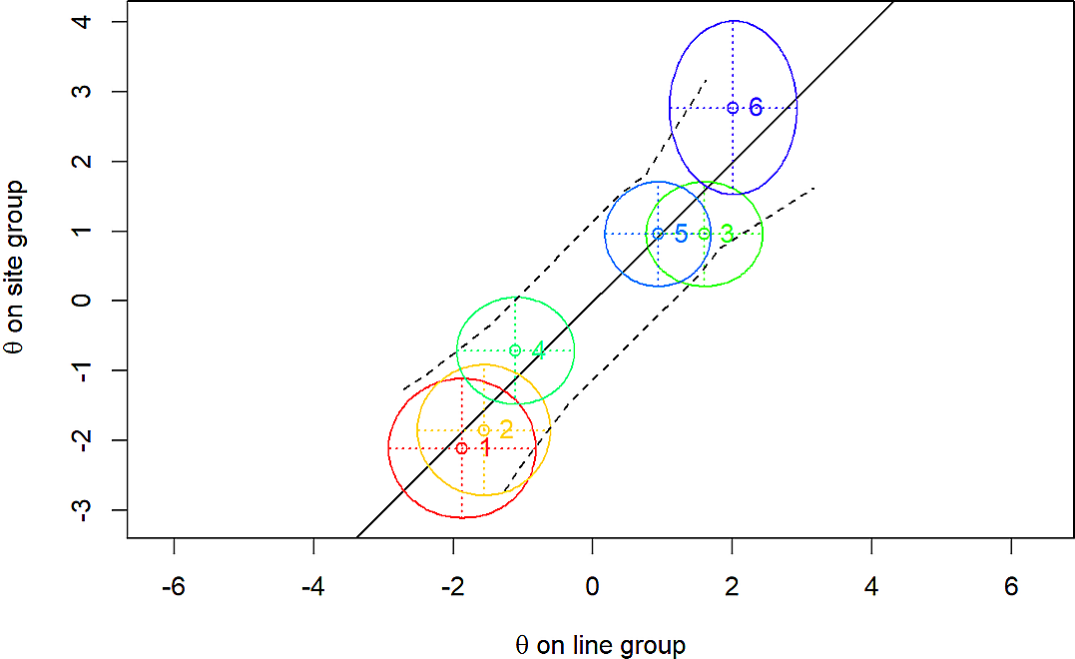
Goodness of fit plot. The numbered colored circles (1-6) represent the location parameters (difficulty) of the 6 items, with circle sizes reflecting the confidence ellipses. The solid diagonal line represents perfect invariance (equal difficulty across groups). The dashed reference lines indicate the 95% confidence bounds. Because all item circles either cross or fall within these dashed boundaries, the item parameters are considered invariant, indicating no significant differential item functioning between the online and onsite student groups.

The nonsignificant T1m statistic in all pairs of questions (*P*>.05) indicated that the practical examination was 1D. Pairwise analysis of item responses (Table S1 in Multimedia Appendix 2) showed no significant violations of unidimensionality, with only a minimal proportion of item pairs displaying residual dependence. The distribution of person fit statistics also showed acceptable fit across participants (Figure 3). The nonsignificant Andersen likelihood ratio test (*χ*^2^_3_=1.2; *P*=.75) confirmed the invariance of the model parameters (Figure 4). Regarding local independence, Ponocny T1 statistic revealed that only the pair “structure identification–patient position” showed a significant residual correlation (*P*=.02). This dependency represented only 4.76% of the total item pairs, a figure within the expected margin of error (<5%) that did not compromise the model’s unidimensionality. This specific correlation was likely due to the procedural nature of the ultrasound examination, in which proper patient positioning serves as a technical prerequisite for successful structure identification. Therefore, this minor violation was considered clinically coherent and statistically negligible for the overall validity of the trait estimates (θ).

#### Item Parameters

**Figure 5.**
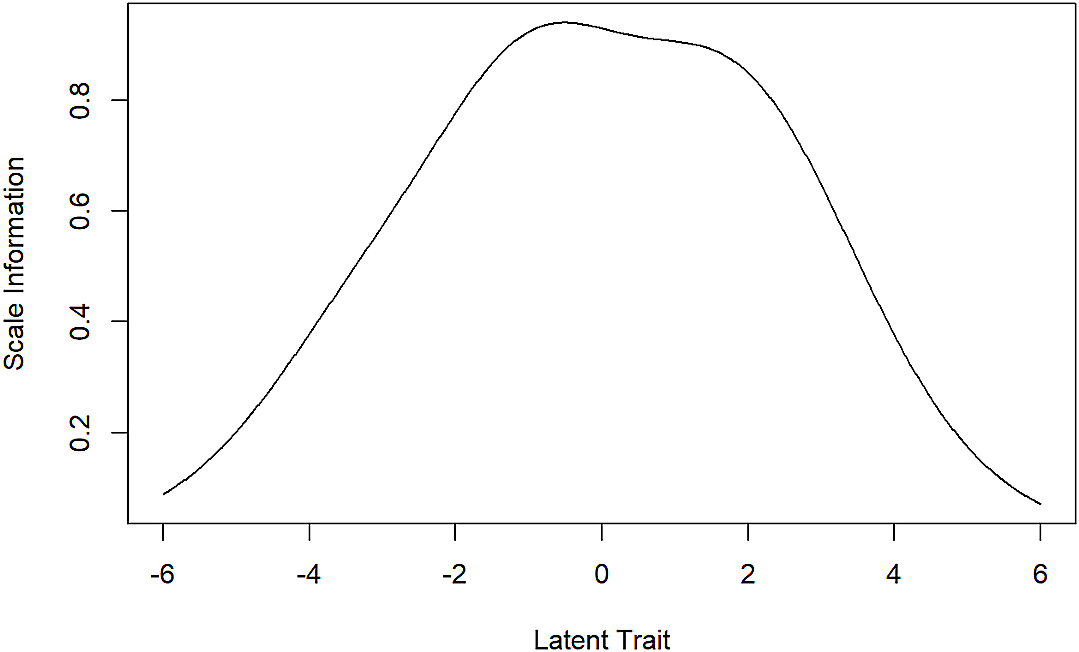
Test information plot illustrating the amount of psychometric information provided by the test across different levels of studentability (latent trait). The curve peaks and remains high between approximately −2 and +2, indicating that the assessment instrument achievesmaximum measurement precision and reliability when evaluating students with average or moderate proficiency levels.

**Figure 6.**
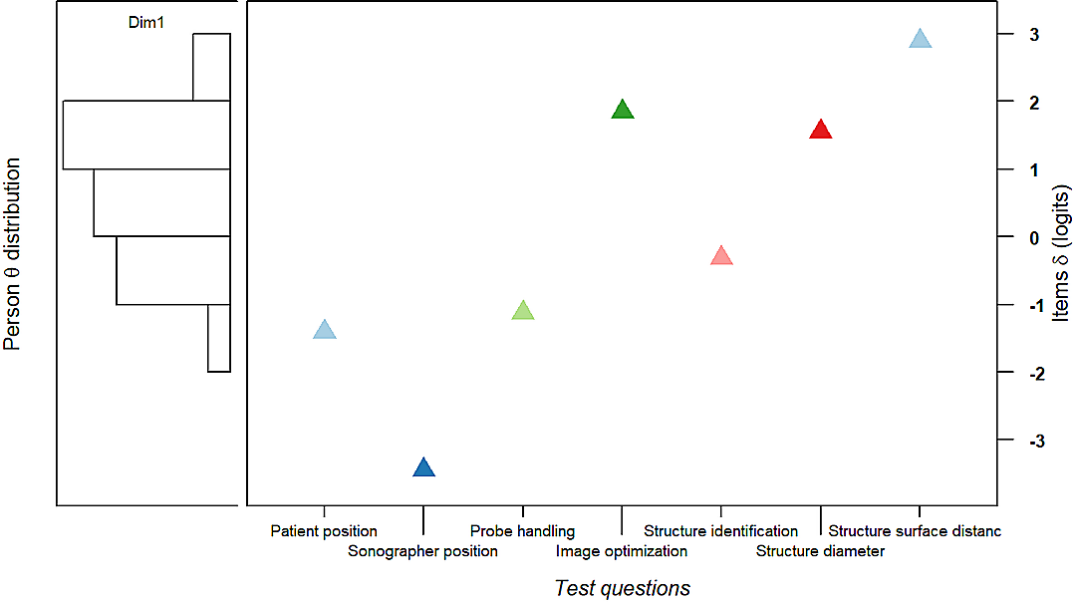
Wright map. The left panel displays a histogram of the participants’ estimated latent proficiency (θ distribution) along the primary latentdimension (Dim1). The right panel plots the difficulty parameters (δ in logits) of the 7 assessment criteria, where each colored triangle representsa specific test question or criterion. Both panels share the central logit scale. Items located higher on the vertical axis (eg, "structure surfacedistance") represent greater difficulty, whereas triangles near the bottom (eg, "sonographer position") indicate easier tasks. This alignment allows direct evaluation of targeting, demonstrating that the test items cover a broad range of the students’ proficiency distribution.

**Figure 7.**
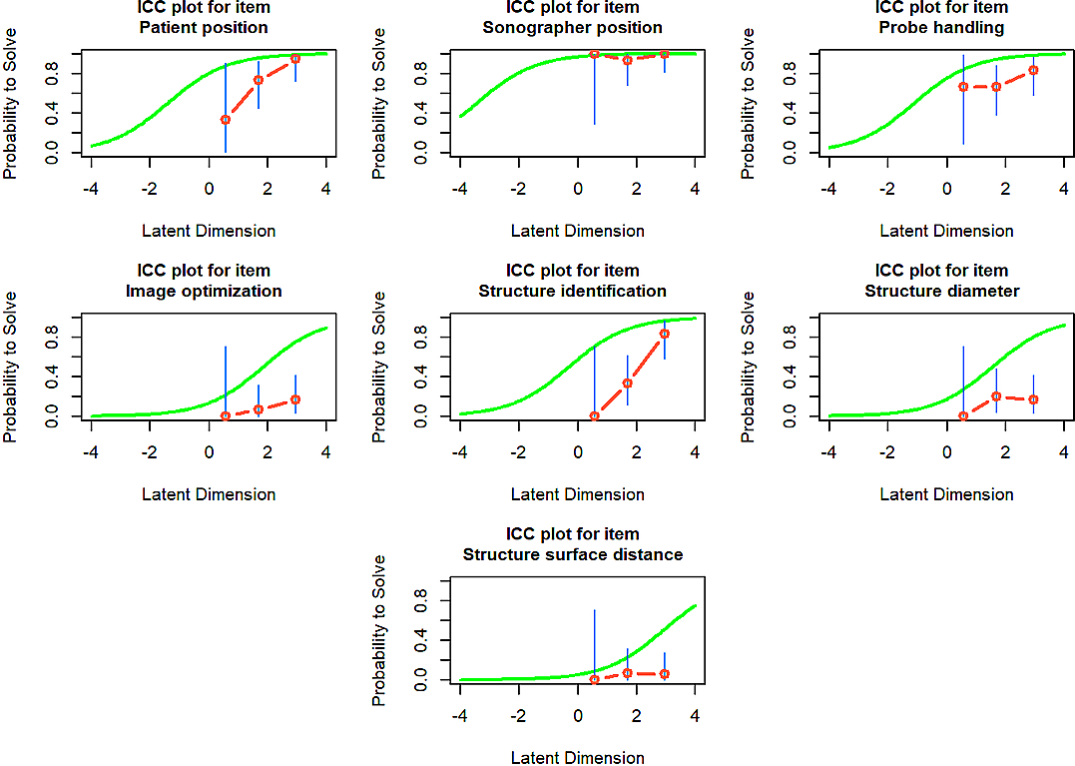
Item characteristic curves. The green solid lines represent the theoretical intraclass correlation coefficient (ICCs; model-predicted probability of a correct response across the latent dimension). The red squares and dashed lines show the observed empirical proportions for each score group, with blue vertical lines indicating the 95% CIs. Closer alignment between the empirical red points and the theoretical green curve reflects better fit of the item to the measurement model.

The examination presented an average level of difficulty δ of 0 (SD 2.213) with a higher difficulty level in the questions on image optimization, structure diameter, and structure surface distance and lower for the questions on patient position, sonographer position, and probe handling (Table 3). It was observed that the test provided the maximum information with the minimum error at levels of θ around −2 to 2 (Figure 5). A clear division of the items was observed according to the level of difficulty; the easiest questions were on patient position and sonographer position; the questions with an intermediate level of difficulty were on probe handling and structure identification; and the most difficult questions were on image optimization, structure diameter, and structure surface distance. The Wright map (Figure 6) illustrated a clear separation between lower- and higher-difficulty items, confirming the hierarchical progression of skill acquisition. The item characteristic curves for the seven assessment criteria are presented in Figure 7, showing the observed response patterns and the model-predicted probabilities across the latent ability continuum.

**Table 3.** Item difficulty parameters.

Item	Difficulty (SE; 95% CI)	
Patient position	−1.407 (0.395; −2.181 to −0.634)	
Sonographer position	−3.446 (0.876; −5.163 to −1.729)	
Probe handling	−1.121 (0.366; −1.837 to −0.405)	
Image optimization	1.845 (0.319; 1.22 to 2.469)	
Structure identification	−0.312 (0.314; −0.928 to 0.303)	
Structure diameter	1.548 (0.308; 0.944 to 2.152)	
Structure surface distance	2.894 (0.399; 2.113 to 3.675)	

## Discussion

### Principal Findings

This randomized study found that simulation-based online ultrasound training produced comparable theoretical and practical outcomes to traditional on-site instruction. IRT analysis revealed a structured hierarchy of item difficulty and identified key competencies for assessment, while both training modalities showed limited reliability relative to expert performance, reflecting the early stage of skill acquisition.

The theoretical and practical assessment outcomes revealed comparable knowledge acquisition across both groups, with no statistically significant differences between the online and on-site training modalities. Notably, the online group demonstrated nearly half the error rate in image identification compared with the on-site cohort, suggesting enhanced visual-cognitive integration when using simulation-based e-learning.

A noteworthy finding was the poor absolute reliability (ICC) and the relatively high SEM and minimal detectable change values observed in both groups. We contextualize these results as a transparent reflection of the learning curve and the expert-novice gap inherent in high-precision technical measurements.

Because the ICC compared student performance against a gold standard expert, these low values underscore that a single training session—regardless of the modality (online or on-site)—is insufficient for achieving clinical-grade proficiency. These data are crucial for future pedagogical designs, as they highlight that while online training might slightly reduce measurement error (SEM), both formats require extended supervised practice to transition from conceptual understanding to technical mastery.

The IRT analysis enabled a detailed understanding of item difficulty within the practical assessment. The practical implications of these findings are 2-fold. First, the item difficulty hierarchy (ẟ) provides a roadmap for curriculum design: training should prioritize “image optimization” and “metric precision” (diameter and distance), as these were identified as the most challenging competencies to acquire (ẟ>1.5). Second, the test information function reveals that the current assessment is highly reliable for discriminating between novice and intermediate students (latent trait between −2 and +2) but may require more challenging items to effectively assess advanced practitioners. Finally, the disparity between observed responses and the ICC curves (Figure 7) in high-difficulty items suggests that metric accuracy is a specific technical bottleneck that persists despite general procedural knowledge, highlighting the need for targeted feedback in ultrasound-guided measurements.

The insights provided by the IRT model allow for a data-driven optimization of the training program, focusing on 3 key areas.

First, regarding curriculum design, because “sonographer position” and “patient position” are low-difficulty items (ẟ<−1.4), they should be introduced as foundational self-learning modules. This allows classroom or simulation time to be shifted toward high-difficulty tasks such as “structure surface distance” (ẟ=2.89), which require expert-led, hands-on guidance.

Second, assessment weighting and IRT difficulty parameters justify a nonlinear scoring system. We suggest that “image optimization” and “metric measurements” should carry higher weight in final assessments, as they are the primary discriminators of technical proficiency, whereas “patient positioning” serves only as a prerequisite.

Third, regarding targeted skill remediation, the item characteristic curves identified specific failure points. For instance, students who failed “structure identification” should not proceed to “structure diameter” training, as the model confirmed a hierarchical dependency. Remediation can thus be personalized based on each student’s position on the latent trait (θ) scale.

No significant differences were found between online and on-site groups in item difficulty patterns. Although ICCs between the 2 groups were nonsignificant and associated with relatively high SEMs, the online group demonstrated slightly better reliability, a finding consistent with previous research [12].

Prior studies have investigated the feasibility of online ultrasound education across various health professions, including obstetrics [9], anesthesia [10], physiotherapy [11,12], and postgraduate echocardiography [13]. These works generally reported equivalent or superior outcomes for knowledge acquisition and image interpretation in comparison to traditional face-to-face methods. These findings are consistent with this body of evidence, supporting the educational validity of simulation-based e-learning for ultrasound training.

However, few previous investigations have objectively assessed psychomotor and image acquisition skills using psychometrically robust frameworks. To our knowledge, no earlier ultrasound education studies have used an IRT model to examine item difficulty and learner ability distribution. This methodological innovation provides a degree of measurement precision rarely applied outside high-stakes simulation domains, such as aviation [20]. Consequently, our results extend the current understanding of how simulation-based training can be objectively evaluated and standardized in medical education.

The findings suggest that digital simulation tools such as the platform can democratize ultrasound training by increasing accessibility and scalability, enabling a broader range of health care professionals to achieve comparable levels of competence. As highlighted by Einstein’s well-known maxim, “doing the same thing and expecting different results” limits innovation [21]; the integration of digital solutions into medical curricula represents a necessary evolution aligned with modern health care and societal needs.

IRT-derived ability mapping indicated that most students clustered at ability levels 1 to 2 within a –3 to +3 range , a valuable reference point for designing adaptive assessment systems. Future studies may leverage these psychometric profiles to personalize training progression and feedback in simulation-based e-learning environments.

Finally, previous evidence demonstrated that online experiential learning models offer significant advantages in efficiency and cost reduction [8]. Combined with comparable learning outcomes, these results reinforce simulation-based digital education as a sustainable and effective approach for future ultrasound and clinical skills training.

Because this study was not prospectively registered, transparency may be limited. Future studies should ensure prior trial registration, use a multi-institution design, and examine a broader cohort.

### Conclusions

Online ultrasound training provides the same theoretical and practical results as face-to-face training, with all the economic, academic, and professional benefits that this entails. The most recommendable questions (“probe handling” and “structure identification”) for the evaluation of this training have been identified. It will be interesting in future studies to evaluate the capacity achieved according to the number of hours, as well as to conduct a cost-effectiveness study.

This study provides a preliminary validation of a practical assessment tool for ultrasound-guided procedures using IRT. The application of the 1-parameter Rasch model proved to be a robust methodological choice for this cohort (n=68); its mathematical parsimony allowed for stable and reliable estimates of item difficulty and student proficiency even in samples of modest size, where more complex models might falter [22,23]. The nonsignificant Andersen likelihood ratio test and the low SEs further reinforced the internal validity of the findings within this specific educational context. However, given the single-site nature of the study and the specific sample size, these results should be interpreted as a foundational pilot validation. While the identified difficulty hierarchy offers a clear road map for curriculum design and targeted remediation, the generalizability and global scalability of the instrument remain to be confirmed. Future research should focus on multicenter studies with larger, more diverse populations to establish definitive normative data and ensure cross-cultural parameter invariance. In conclusion, this work represents a significant step toward a data-driven, standardized evaluation in ultrasound training, providing a reliable framework for initial competency assessment.

## Supplementary material

10.2196/87897 Multimedia Appendix 1 Detailed item response theory analysis and model diagnostics.

10.2196/87897 Multimedia Appendix 2 Psychometric analyses and graphical outputs from the item response theory (IRT) Rasch model, including item characteristic curves, Wright maps, goodness-of-fit plots, and test information functions comparing online and on-site ultrasound training groups.

## Abbreviations

CONSORT: Consolidated Standards of Reporting Trials
ICC: intraclass correlation coefficient
IRT: item response theory
SEM: standard error of measurement
