# Supplementary material for: Digital Simulation–Based Ultrasound Training for Physiotherapy Students: Prospective Randomized Controlled Trial
Source: JMIR Med Educ. 2026 Jul 2;12:e87897. doi: 10.2196/87897 (PMC13327370; doi:10.2196/87897)
Supplement: Multimedia Appendix 1 [file mededu-v12-e87897-s001.docx]

# Multimedia Appendix 1. Detailed Item Response Theory Analysis and Model Diagnostics

## Overview

This appendix provides a comprehensive description of the Item Response Theory (IRT) methodology and detailed statistical results supporting the findings reported in the main manuscript.

## IRT Model Assumptions

The Rasch model was applied under the assumptions of unidimensionality, local independence, and parameter invariance. Unidimensionality was assessed using the T1m statistic, while local independence was evaluated using the T1 statistic. Parameter invariance was tested using Andersen’s Likelihood Ratio Test.

For the IRT analysis, the questions Structure diameter, Structure surface distance were dichotomized as correct/incorrect based on presenting a value within the limit of ±10% of the value of reference. A Rasch model was used to calculate the level of difficulty (δ) of the items and the level of ability (θ) of the students. Due to the small size of the sample, the non-parametric tests proposed by Ponocny et al. (2001)[1] and Koller et al. (2013)[2] to evaluate compliance with the assumptions of unidimensionality (T1m statistic) and local independence (T1 statistic) while the invariance of the parameters was evaluated

Andersen´s Likelihood Ratio Test. Item and person fit were assessed by analyzing the infit and outfit values for both items and students. For the mean-square statistics tolerance limits of 0.5–1.5 were adopted[2]. Additionally, a threshold of ±2 was used for the standardized t statistics(infit and outfit)[3]. The rationale for this threshold is that t statistics are transformed to approximate a unit normal distribution (mean = 0, SD = 1); therefore, values exceeding ±2 indicate a statistically significant departure from the model’s expectations (p < .05). This combined approach ensures that the response patterns are consistent with the Rasch model's requirements for unidimensionality and stability. Model fit was assessed through the significance level of the Hosmer-Lemershow test, R2 value, Area Under the Curve (AUC), sensitivity and specificity. Homogeneity between groups was assessed using the non-parametric T10 statistic and the Wald test. Differential Item Functioning (DIF) was analyzed based on group and gender using the non-parametric statistic T4[17,20].

## Model Fit and Diagnostics

Model fit was evaluated using multiple complementary approaches:

- Andersen’s Likelihood Ratio Test
- Hosmer-Lemeshow goodness-of-fit test
- Infit and outfit mean-square statistics
- Standardized t statistics (threshold ±2)

The Hosmer-Lemeshow test indicated good model fit (p>0.05). Infit and outfit values were interpreted within the acceptable range of 0.5–1.5.

## Item Parameters

Item difficulty parameters (δ) were estimated for all practical exam items. Items related to image optimization and metric measurements (structure diameter and surface distance) showed higher difficulty levels, whereas patient positioning and probe handling demonstrated lower difficulty.

## Person Parameters

Student ability (θ) estimates showed a distribution primarily between -2 and +2, indicating that the test was most informative for distinguishing novice to intermediate learners.

## Reliability and Agreement

Interobserver reliability was assessed using Intraclass Correlation Coefficients (ICC), Standard Error of Measurement (SEM), and Minimal Detectable Change (MDC). Detailed values for each measurement are provided in Supplementary Tables.

## Differential Item Functioning

Differential Item Functioning (DIF) analysis was conducted by group and gender using the T4 statistic. No significant DIF was detected.

## Additional Tables and Figures

The following materials are included in this appendix:

- Full item difficulty table
- Detailed ICC, SEM, and MDC results
- Item Characteristic Curves
- Wright map
- Test Information Function
- Model fit plots and residual analyses

These additional materials support the robustness of the IRT model and provide transparency for reproducibility.

References

1. Ponocny, I. Nonparametric goodness-of-fit tests for the Rasch model. *Psychometrika* **2001**, *66*, 437–459, doi:10.1007/BF02294444/METRICS.

2. Koller, I.; Hatzinger, R. Nonparametric tests for the Rasch model : explanation , development , and application of quasi-exact tests for small samples. **2013**.

3. Hambleton, R.K.; Swaminathan, H. Assumptions of Item Response Theory. *Item Response Theory* **1985**, 15–31, doi:10.1007/978-94-017-1988-9_2.
