# Supplementary material for: Digital Simulation–Based Ultrasound Training for Physiotherapy Students: Prospective Randomized Controlled Trial
Source: JMIR Med Educ. 2026 Jul 2;12:e87897. doi: 10.2196/87897 (PMC13327370; doi:10.2196/87897)
Supplement: Multimedia Appendix 2 [file mededu-v12-e87897-s002.docx]

Digital Simulation–Based Ultrasound Training in Physiotherapy Students: Blinded Randomized Controlled Trial Applying Item Response Theory

| Supplementary material. Table 1: Item-pairs comparison. | | |
| --- | --- | --- |
|  | **T1m statistic^a^** | **T1 statistic^a^** |
| Image optimization - Patient position | 0.89 | 0.54 |
| Image optimization - Probe handling | 0.9 | 0.36 |
| Image optimization - Sonographer position | >0.999 | 0.78 |
| Probe handling - Patient position | 0.31 | >0.999 |
| Probe handling - Sonographer position | >0.999 | 0.11 |
| Sonographer position - Patient position | 0.88 | >0.999 |
| Structure diameter - Image optimization | 0.28 | 0.93 |
| Structure diameter - Patient position | 0.3 | 0.93 |
| Structure diameter - Probe handling | 0.74 | 0.5 |
| Structure diameter - Sonographer position | >0.999 | 0.71 |
| Structure diameter - Structure identification | 0.52 | 0.75 |
| Structure identification - Image optimization | 0.47 | 0.81 |
| Structure identification - Patient position | 0.99 | 0.02 |
| Structure identification - Probe handling | 0.53 | 0.86 |
| Structure identification - Sonographer position | 0.63 | >0.999 |
| Structure surface distance - Image optimization | 0.11 | >0.999 |
| Structure surface distance - Patient position | >0.999 | 0.44 |
| Structure surface distance - Probe handling | 0.26 | 0.96 |
| Structure surface distance - Sonographer position | 0.08 | >0.999 |
| Structure surface distance - Structure diameter | 0.67 | 0.71 |
| Structure surface distance - Structure identification | >0.999 | 0.17 |
| ^a^significant if p<0.05 (Shown in red). |  |  |

| Supplementary material. Table 2: Person ability and infit-oufit statistics. | | | | | |
| --- | --- | --- | --- | --- | --- |
|  | **Ability (SE)** | **95%CI** | **Infit** | **Outfit** |  |
| 1 | 0.577 (SE=1.047) | -1.474, 2.629 | 1.529 | 1.293 |  |
| 2 | 1.687 (SE=1.066) | -0.402, 3.775 | 1.261 | 0.799 |  |
| 3 | 0.577 (SE=1.047) | -1.474, 2.629 | 0.28 | 0.215 |  |
| 4 | 1.687 (SE=1.066) | -0.402, 3.775 | 0.569 | 0.324 |  |
| 5 | 0.577 (SE=1.047) | -1.474, 2.629 | 1.604 | 1.558 |  |
| 6 | 0.577 (SE=1.047) | -1.474, 2.629 | 1.935 | 2.405 |  |
| 7 | 1.687 (SE=1.066) | -0.402, 3.775 | 1.261 | 0.799 |  |
| 8 | 0.577 (SE=1.047) | -1.474, 2.629 | 0.28 | 0.215 |  |
| 9 | -0.5 (SE=1.031) | -2.522, 1.521 | 0.882 | 0.54 |  |
| 10 | 2.95 (SE=1.23) | 0.54, 5.361 | 0.555 | 0.244 |  |
| 11 | 1.687 (SE=1.066) | -0.402, 3.775 | 0.569 | 0.324 |  |
| 12 | 1.687 (SE=1.066) | -0.402, 3.775 | 0.737 | 0.409 |  |
| 13 | 2.95 (SE=1.23) | 0.54, 5.361 | 1.272 | 0.612 |  |
| 14 | 1.687 (SE=1.066) | -0.402, 3.775 | 0.737 | 0.409 |  |
| 15 | 1.687 (SE=1.066) | -0.402, 3.775 | 0.569 | 0.324 |  |
| 16 | -0.5 (SE=1.031) | -2.522, 1.521 | 0.882 | 0.54 |  |
| 17 | 0.577 (SE=1.047) | -1.474, 2.629 | 0.28 | 0.215 |  |
| 18 | 2.95 (SE=1.23) | 0.54, 5.361 | 1.272 | 0.612 |  |
| 19 | 0.577 (SE=1.047) | -1.474, 2.629 | 0.28 | 0.215 |  |
| 20 | 0.577 (SE=1.047) | -1.474, 2.629 | 0.28 | 0.215 |  |
| 21 | 0.577 (SE=1.047) | -1.474, 2.629 | 0.28 | 0.215 |  |
| 22 | 1.687 (SE=1.066) | -0.402, 3.775 | 0.737 | 0.409 |  |
| 23 | 2.95 (SE=1.23) | 0.54, 5.361 | 0.555 | 0.244 |  |
| 24 | 0.577 (SE=1.047) | -1.474, 2.629 | 0.28 | 0.215 |  |
| 25 | 1.687 (SE=1.066) | -0.402, 3.775 | 0.569 | 0.324 |  |
| 26 | 0.577 (SE=1.047) | -1.474, 2.629 | 0.28 | 0.215 |  |
| 27 | 1.687 (SE=1.066) | -0.402, 3.775 | 0.569 | 0.324 |  |
| 28 | 1.687 (SE=1.066) | -0.402, 3.775 | 0.569 | 0.324 |  |
| 29 | 1.687 (SE=1.066) | -0.402, 3.775 | 0.569 | 0.324 |  |
| 30 | 1.687 (SE=1.066) | -0.402, 3.775 | 0.569 | 0.324 |  |
| 31 | 0.577 (SE=1.047) | -1.474, 2.629 | 0.28 | 0.215 |  |
| 32 | 0.577 (SE=1.047) | -1.474, 2.629 | 0.28 | 0.215 |  |
| 33 | 1.687 (SE=1.066) | -0.402, 3.775 | 1.261 | 0.799 |  |
| 34 | 0.577 (SE=1.047) | -1.474, 2.629 | 0.28 | 0.215 |  |
| 35 | 0.577 (SE=1.047) | -1.474, 2.629 | 1.65 | 1.437 |  |
| 36 | -0.5 (SE=1.031) | -2.522, 1.521 | 0.882 | 0.54 |  |
| 37 | -0.5 (SE=1.031) | -2.522, 1.521 | 1.014 | 0.648 |  |
| 38 | 1.687 (SE=1.066) | -0.402, 3.775 | 0.737 | 0.409 |  |
| 39 | 1.687 (SE=1.066) | -0.402, 3.775 | 0.737 | 0.409 |  |
| 40 | 1.687 (SE=1.066) | -0.402, 3.775 | 0.569 | 0.324 |  |
| 41 | 2.95 (SE=1.23) | 0.54, 5.361 | 0.555 | 0.244 |  |
| 42 | -0.5 (SE=1.031) | -2.522, 1.521 | 2.834 | 7.502 |  |
| 43 | 1.687 (SE=1.066) | -0.402, 3.775 | 0.737 | 0.409 |  |
| 44 | 1.687 (SE=1.066) | -0.402, 3.775 | 0.737 | 0.409 |  |
| 45 | 1.687 (SE=1.066) | -0.402, 3.775 | 0.737 | 0.409 |  |
| 46 | 1.687 (SE=1.066) | -0.402, 3.775 | 0.737 | 0.409 |  |
| 47 | -0.5 (SE=1.031) | -2.522, 1.521 | 0.462 | 0.297 |  |
| 48 | -0.5 (SE=1.031) | -2.522, 1.521 | 0.462 | 0.297 |  |
| 49 | -0.5 (SE=1.031) | -2.522, 1.521 | 0.462 | 0.297 |  |
| 50 | -0.5 (SE=1.031) | -2.522, 1.521 | 1.735 | 1.683 |  |
| 51 | 1.687 (SE=1.066) | -0.402, 3.775 | 1.523 | 1.405 |  |
| 52 | -0.5 (SE=1.031) | -2.522, 1.521 | 1.792 | 2.071 |  |
| 53 | -0.5 (SE=1.031) | -2.522, 1.521 | 0.462 | 0.297 |  |
| 54 | -0.5 (SE=1.031) | -2.522, 1.521 | 0.462 | 0.297 |  |
| 55 | 0.577 (SE=1.047) | -1.474, 2.629 | 1.23 | 0.827 |  |
| 56 | -0.5 (SE=1.031) | -2.522, 1.521 | 1.735 | 1.683 |  |
| 57 | -1.597 (SE=1.083) | -3.72, 0.526 | 0.767 | 0.422 |  |
| 58 | 0.577 (SE=1.047) | -1.474, 2.629 | 1.351 | 0.972 |  |
| 59 | -0.5 (SE=1.031) | -2.522, 1.521 | 0.462 | 0.297 |  |
| 60 | -0.5 (SE=1.031) | -2.522, 1.521 | 1.603 | 1.575 |  |
| 61 | -1.597 (SE=1.083) | -3.72, 0.526 | 0.767 | 0.422 |  |
| 62 | -1.597 (SE=1.083) | -3.72, 0.526 | 0.604 | 0.336 |  |
| 63 | 0.577 (SE=1.047) | -1.474, 2.629 | 1.351 | 0.972 |  |
|  | | | | |  |

| Supplementary material. Table 2: Wald test by item. | | |
| --- | --- | --- |
|  | **Z statistic** | **^a^p value** |
| Patient position | -0.322 | .747 |
| Probe handling | -0.423 | .672 |
| Image optimization | -1.124 | .261 |
| Structure identification | 0.674 | .5 |
| Structure diameter | 0.041 | .967 |
| Structure surface distance | 0.961 | .336 |
| ^a^significant if p<.05 (Shown in red). |  |  |

- Person parameters: It is verified how the ability level θ of the students increases linearly with the total score of the test (Supplementary material. Table 1).
- Person and item fit: Items Patient position, Sonographer position present outfit values outside the tolerance limit of 0.5-1.5, which indicates problems in their adjustment (Table 4 were Values lower than 0.5 and upper 1.5 shown in red). The graph of items vs. infit t statistic shows how the questions Sonographer position, Structure surface distance present values above the tolerance limit of ±2, which indicates problems in their fit to the model (Main Manuscript. Figure 1 ). A total of 27 students present infit values less than 0.5 or greater than 1.5 and 48 students outfit values lower than 0.5 or higher than 1.5, which indicates an inadequate adjustment of the same. This suggests that students' responses were highly deterministic and followed the established difficulty hierarchy with high consistency. Rather than indicating a flaw in the model, this overfit reinforces the stability of the construct, showing that foundational skills are consistently mastered before advanced ones. The isolated cases of person-underfit (Outfit > 1.5) likely reflect the non-linear nature of practical skill acquisition. In ultrasound training, even proficient students may occasionally fail foundational tasks (e.g., patient positioning) due to the high cognitive demand of simultaneously performing complex technical measurements. These minor inconsistencies, often exacerbated by the dichotomization of performance variables, represent individual clinical variability and do not compromise the overall scalability of the assessment instrument (Supplementary material. Table 2). The graph of students vs. infit t statistic shows how the majority presents values within the tolerance limit of ±2, which indicates an adequate fit to the model (Main Manuscript. Figure 8).

| Table 4: Items infit-outfit statistics. | | |
| --- | --- | --- |
|  | **Infit** | **Outfit** |
| Patient position | 0.703 | 0.443 |
| Sonographer position | 0.661 | 0.328 |
| Probe handling | 0.882 | 0.62 |
| Image optimization | 0.953 | 0.885 |
| Structure identification | 0.724 | 0.62 |
| Structure diameter | 0.959 | 0.988 |
| Structure surface distance | 0.838 | 0.998 |
| ^a^significant if p<0.05 (Shown in red). |  |  |

- Model fit: Hosmer-Lemershow test not significant (X^2^(8) =7.251, p=0.51) indicates a good model fit. The model has a sensitivity of 87.452% and a specificity of 80.337% with an area under the curve (AUC) of 92.626%, all of which are excellent values. The variance explained by the model is moderate (R^2^=0.557). The non-significant T10 statistic (p=0.91) indicates the presence of homogeneity between both groups with a good fit of the model. The non-significant Wald test for each question again indicates the presence of homogeneity between the two groups in the responses to each item (significant differences are shown in red) (Supplementary material. Table 2). The fit plot shows how the items are evenly distributed within the approximate range of θ of ±2, the overlap in the areas of the ellipses in the items Patient position, Probe handling, Structure identification and Image optimization, Structure diameter, Structure surface distance indicate that both sets of questions cover the same θ level of students (Main Manuscript. Figure 5).
- DIF (differential) analysis: The non-significant T4 statistic for both group (p=0.58) and gender (0.44) indicate the absence of DIF.
